# Multi-modal pain measurements in infants

**DOI:** 10.1016/j.jneumeth.2012.01.009

**Published:** 2012-04-15

**Authors:** A. Worley, L. Fabrizi, S. Boyd, R. Slater

**Affiliations:** a Department of Clinical Neurophysiology, Great Ormond Street Hospital for Children, London WC1N 3JH, UK; b Department of Neuroscience, Physiology and Pharmacology, University College London, London WC1E 6BT, UK; c Nuffield Department of Anaesthetics, University of Oxford, Oxford OX3 9DU, UK

**Keywords:** Human infants, Noxious stimulation, Tactile stimulation, Pain, Multi-modal recordings, Neural responses

## Abstract

A non-invasive integrated method was developed to measure neural and behavioural responses to peripheral sensory and noxious stimulation in human infants. The introduction of a novel event-detection interface allows synchronous recording of: (i) muscle and central nervous system activity with surface electromyography (EMG), scalp electroencephalography (EEG) and near-infrared spectroscopy (NIRS); (ii) behavioural responses with video-recording and (iii) autonomic responses (heart rate, oxygen saturation, respiratory rate and cardiovascular activity) with electrocardiography (ECG) and pulse oximetry. The system can detect noxious heel lance and touch stimuli with precision (33 μs and 624 μs respectively) and accuracy (523 μs and 256 μs) and has 100% sensitivity and specificity for both types of stimulation. Its ability to detect response latencies accurately was demonstrated by a shift in latency of the vertex potential of 20.7 ± 15.7 ms (*n* = 6 infants), following touch of the heel and of the shoulder, reflecting the distance between the two sites. This integrated system has provided reliable and reproducible measurements of responses to sensory and noxious stimulation in human infants on more than 100 test occasions.

## Highlights

► We describe a method to measure neural responses to noxious stimulation in infants. ► Multi-modal recordings (EEG, EMG, ECG, NIRS) are synchronised within this method. ► The method meets ethical and safety standards. ► The method incorporates a novel event-locking interface to a disposable medical device. ► We have successfully used the method on more than 100 test occasions.

## 1 Introduction

Pain is a human experience, of which we all have intuitive understanding. A definition of pain, widely used in nursing, states that “pain is whatever the experiencing person says it is, existing whenever he says it does” (McCaffrey and Pasero, 1999). Necessarily, from this definition, self-reporting is crucial for accurate pain diagnosis, but infants’ lack of language renders this impossible. On the other hand, the understanding and management of pain in this population is as important, if not more so, than in adults because early pain experiences may cause long term disruptive alterations of sensory perception (Fitzgerald and Walker, 2009). It is therefore fundamental to devise alternative, even if indirect, techniques of pain assessment, which do not require self-report.

In current clinical practice infant pain is usually inferred from observations of behavioural and autonomic activity. For example, changes in facial expression, crying, heart rate and oxygen saturation form the basis of a number of pain scales and scores (Duhn and Medves, 2004). However, in the developing central nervous system (CNS) where pain pathways are maturing and neural processing is developing, the correspondence between pain perception and behavioural response is unknown (Slater et al., 2008). The observed changes in behavioural and autonomic responses to nociception are mediated by subcortical structures and therefore provide indirect measures of pain, which is known to involve sensory-discriminative, negative affective and motivational processing at cortical level (Tracey and Mantyh, 2007; Treede et al., 1999).

In the study of pain the term *nociception* denotes the neural processes of encoding and processing noxious stimuli. It refers to the afferent activity in the peripheral and central nervous system evoked by a stimulus that causes or has the potential to cause tissue damage (IASP Task Force on Taxonomy, 1994). For an experience to be painful, it requires that the noxious signal is transmitted to the cortex (Tracey and Mantyh, 2007). It is therefore plausible that measuring nociceptive activity from the CNS – although clearly still a surrogate measure of pain perception – is a more direct way of quantifying pain in newborn infants compared to observing behavioural and autonomic activity. This notion is supported by studies in adult volunteers which show that the size of the cortical activity evoked by a noxious stimulation is directly related to the intensity of the stimulus and verbally reported pain (Bromm and Scharein, 1982; Iannetti et al., 2005). By using such an approach in infants, it is possible that the many advances that have been made in the understanding of adult pain can be translated to the paediatric population. Cortical activity also lends itself more naturally to objective, quantitative measurement than many of the behavioural indicators, and has the advantage that there are clear parallels in animal models which allow laboratory investigation of the underlying physiology and pharmacology (Rainville, 2002; Schnitzler and Ploner, 2000; Takeda et al., 2010).

In this paper we describe a non-invasive and ethically acceptable technique to simultaneously measure the traditionally observed behavioural and autonomic responses together with newer electrophysiological and haemodynamic measures of CNS activity to provide an integrative approach to the study of pain in the neonatal population. While it is relatively simple to undertake laboratory experiments on adult volunteers using laser, electrical, thermal and mechanical noxious stimuli, regulatory and ethical considerations rightly exclude these possibilities in the study of pain in newborn infants. In this respect, the clinically required blood sampling procedure used to extract blood from hospitalised infants offers a feasible and unique opportunity to study neonatal pain. Infants routinely have their heels lanced with a spring-loaded blade so that blood samples can be extracted for clinical analysis. This represents a clinically essential acute noxious peripheral input that can be used to study infant responses to this type of stimulation. On these occasions, flexion reflex withdrawal activity can be recorded using surface electromyography (EMG); brain activity can be recorded with scalp electroencephalography (EEG) and near-infrared spectroscopy; behavioural responses can be monitored using video-recording; and autonomic responses (including heart rate, oxygen saturation, respiratory rate and cardiovascular activity) can be recorded using electrocardiography (ECG) and pulse oximetry. By designing a time-locking interface to synchronise these measurements with noxious heel lances and other types of control stimulations (e.g. non-noxious touch) it has been possible to develop a fully integrated method to measure evoked responses to noxious events in newborn infants.

## 2 Methods

### 2.1 Physiological recording techniques

#### 2.1.1 Neuronal brain activity: electroencephalography (EEG)

Seventeen recording Ag/AgCl cup electrodes are positioned according to the international 10/20 electrode placement system. Reference and ground electrodes are placed at FCz and the chest/head respectively. Electrode/skin impedance is kept to a minimum by rubbing the skin with an EEG prepping gel. Conductive EEG paste is used to optimise contact with the electrodes. Electrodes are held in place by an elastic bandage and leads are tied together to minimise electrical interference. Electrodes are disposed of after each study. EEG activity, from DC to 70 Hz, is recorded using the Neuroscan SynAmps2 EEG/EP recording system (Compumedics Ltd.). Signals are sampled at a rate of 2 kHz and subsequently digitised with a resolution of 24 bit.

#### 2.1.2 Haemodynamic brain activity: near-infrared spectroscopy (NIRS)

Cortical activity is associated with increased localised cerebral blood flow. A double-channel near-infrared spectrophotometer (NIRO-200; Hamamatsu Photonics) is used to measure regional changes in oxygenated and deoxygenated haemoglobin concentration. The light emitters and detectors (optodes) are positioned symmetrically on either side of the head over the somatosensory cortex, using the international 10–20 EEG placement system to identify key landmarks. Changes in oxyhaemoglobin [HbO2] and deoxyhaemoglobin [HHb] are measured in real time. Total haemoglobin [HbT] concentration change can be calculated as [HbT] = [HbO2] + [HHb].

#### 2.1.3 Withdrawal reflex activity: electromyography (EMG)

Surface electromyography (EMG) is recorded from the biceps femoris muscle of both the stimulated and unstimulated leg. EMG signals are recorded using bipolar channel recording. The surface of the skin is rubbed with an EEG prepping gel. Self-adhesive surface electrodes are positioned over the muscle belly and held in place with self-adherent elastic wrap. Leg motion is recorded with a movement transducer secured to the leg. The signal is grounded at the chest. EMG activity is recorded from 10 to 500 Hz and sampled at 2 kHz using the EEG recording system. A 50 Hz notch filter can be applied when required.

#### 2.1.4 Behavioural activity: video monitoring

Infant behavioural activity is monitored using a tripod-mounted video recorder. The videos are analysed off-line to characterise change in facial expression, to assess the sleep state and detect behavioural activity such as, facial twitching, sucking and, eye and limb movements.

#### 2.1.5 Autonomic activity: pulse oximetry, electrocardiography (ECG), respiration

Autonomic activity is measured by recording heart rate, oxygen saturation, respiratory rate and cardiovascular activity. Heart rate and oxygen saturations recorded using a pulse oximeter (NellcorOximax N-560). Lead I ECG is used to record ECG activity and a movement transducer placed on the abdomen records the respiration through the EEG headbox.

### 2.2 Event-detection interface

The physiological recordings are acquired concurrently and synchronised to the sensory events through a custom-designed event-detection interface. The interface detects the occurrence of the stimulations and event-marks the recordings simultaneously. The interface has to have sensitive and specific detection for each sensory event so that precise and accurate marking can ensure that measured variation in the response latencies is due to physiological variability and not measurement error. Moreover, the designed system must not interfere with the infant's clinical requirements, and must comply with the infection control protocols on the intensive or special care unit of the hospital.

#### 2.2.1 Sensory event detection

##### 2.2.1.1 Noxious heel lance

**Fig. 1:**
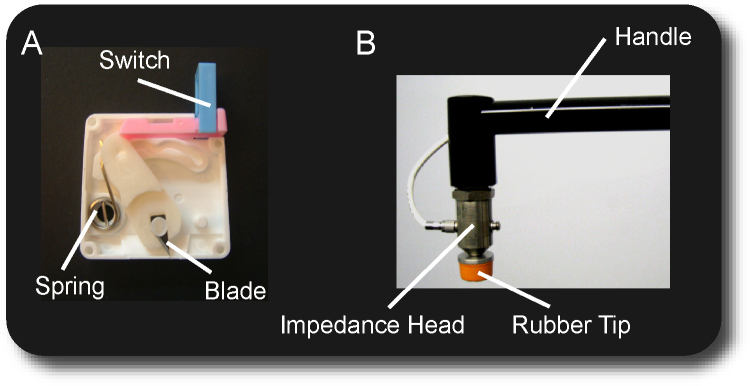
(A) Lancet used for the heel lance; (B) modified tendon hammer used for tactile stimulation.

The lancet used for the heel lance is a sterile disposable automatic incision device (Tenderfoot, International Technidyne Corporation). The lancet houses a spring-loaded surgical blade that is released when a small switch on the superior surface is depressed. When the device is activated, the blade swings in an arc such that for a short time the blade protrudes from the device and makes an incision in the superficial layers of the skin. After the blade is released it automatically and permanently retracts (Fig. 1A).

**Fig. 2:**
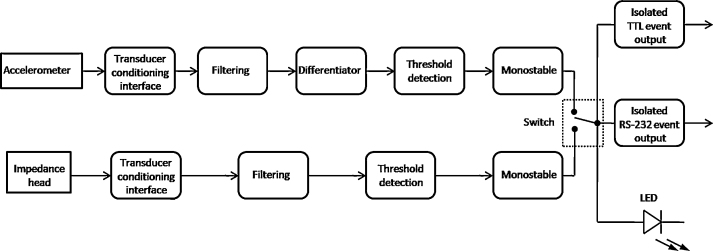
Block diagram of the event detection interface used for detecting blade release from the lancet (upper section), or the delivery of tactile stimuli (lower section). Mode of event marking is determined by the switch.

The releasing of the blade causes a characteristic vibration of the lancet. In order to detect this event, an accelerometer (K-shear type, Kistler Instruments Ltd.) is attached to the superior surface of the lancet. The output of the accelerometer is fed to a discriminator circuit calibrated to identify the characteristic vibration. This mainly consists of a differentiator circuit followed by a Schmitt trigger and a monostable (Fig. 2). The threshold for the Schmitt trigger was set by observing the derivative of the accelerometer's output generated by triggering 10 lances.

##### 2.2.1.2 Non-noxious touch

Non-noxious touch is performed with a modified tendon hammer, which is used to tap the heel or other sites of the body (the shoulder in this present study). An impedance head with a built-in calibrated force transducer (Bruel & Kjaer, Type 8001, Denmark) is placed between the handle and the rubber tip of the tendon hammer (Fig. 1B). This measures the force applied and determines the time of stimulation. In order to do this, the output of the force transducer is processed by a similar discriminator circuit as described earlier (Fig. 2). The threshold for event marking was set by measuring the output of the force transducer when exerting the range of forces used for non-noxious stimulation (25.5 ± 7.5 N).

#### 2.2.2 Event marking of the recordings

The output of the event-detection interface (for both the noxious and non-noxious stimulation) is transmitted to the digital input (‘trigger’) ports of the physiological recording devices. With the exclusion of the video recording, all the other data are downloaded in real time on a host computer. The Neuroscan SynAmps2 data, including the event mark provided by the detection interface are downloaded using the companion software Scan 4.3 (Compumedics US Co.) through a USB 2.0 interface. The OxiMax N-560 data are downloaded using a custom MATLAB (The Mathworks, USA) software application through a RS232 link alongside a 1 bit input from the parallel port which is used to record the event mark. The NIRO-200 data are downloaded using its digital output (RS232) and associated software. The outputs of the monostables of the event-detection interface drive: (i) a TTL pulse generator to mark the event on the Neuroscan SynAmps2 (i.e. EEG, EMG, ECG, respiration) through the external digital input of the recording system and on the Oximax N-560 through the parallel port; (ii) an RS232 link to mark the event on the NIRO-200 and (iii) a LED flash to mark the event on video.

### 2.3 Functional evaluation

#### 2.3.1 Event detection precision and accuracy

The precision and accuracy of the event-detection interface was tested adopting an optical infrared measurement system. This consisted of a transmitter photo-diode and a matched photo-transistor receiver with 5 μs temporal resolution (Maplin Electronics, CH10L and CH11M) placed 5 cm apart. Ten lancets were triggered such that the blade, when released, interrupted the infrared beam. The TTL output of the event-detection interface was compared with the time at which the blade interrupted the infrared beam on a fast sampling oscilloscope (Fluke Instruments, Model 196C, sampling rate 1GS/s). A similar experiment was repeated by tapping the surface between the photodiode and transistor with the tendon hammer on 10 occasions, and comparing the TTL output of the event-detection interface with the time at which the tip of the hammer interrupted the infrared beam.

#### 2.3.2 Event detection sensitivity and specificity

The event-detection interface should produce a change in output signal only in the event of a planned stimulation and not by merely handling the lancet or the tendon hammer. To test this capability, event detection sensitivity and specificity were evaluated. A lancet and the tendon hammer were waved 20 times to estimate the false positive (FP) and true negative (TN) rates. These were compared with the true positive (TP) and false negative (FN) rates estimated by simulating 20 real stimulations by triggering 20 lancets and tapping the tendon hammer on the forearm 20 times.

#### 2.3.3 Clinical validation of the system

Clinical tests were conducted to check the feasibility of the designed integrated method to simultaneously record synchronised CNS, autonomic and behavioural responses following tactile stimulation and noxious lance of the heel.

The reliability of event detection was assessed following tactile stimulation of the heel and of the shoulder of 6 newborn infants. These were recruited from the special care baby unit at the Elizabeth Garrett Anderson and Obstetric Hospital. Ethical approval was obtained from the UCLH Ethics Committee and informed written parental consent was obtained prior to each study. The study conformed to the standards set by the Declaration of Helsinki.

Peripheral tactile stimulation elicits an event related potential (ERP) maximal at the vertex (CPz) in the EEG recording (Hrbek et al., 1968, 1973; Slater et al., 2010). Infants were tapped 8 ± 4 times on the heel and on the shoulder with an inter stimulus interval of 12.8 ± 5.7 s. EEG epochs including activity recorded at CPz from 500 ms before to 1000 ms after each event mark were considered. Epochs recorded within each infant were averaged for heel and shoulder stimulation separately. The mean time delay between the ERP evoked by touching the heel and the shoulder was then calculated to check whether the system is capable of reliably detecting such latency differences.

## 3 Results

### 3.1 Event detection precision and accuracy

The release of the blade interrupted the infrared beam for 1.73 ± 0.1 ms (mean ± SD; range: 1.6–1.9 ms). The event-detection was timed to the middle of this interval as this corresponds to the time at which the blade is at its maximal depth in the infant foot. The timing of the lance could be determined with a precision of 33 μs and an accuracy of 523 μs (offset range: −900 to 500 μs).

The timing of the touch could be determined with a precision of 624 μs and an accuracy of 256 μs (offset range: −1100 to −160 μs).

### 3.2 Event detection sensitivity and specificity

Waving the lancet and the tendon hammer did not cause any false event mark (FP = 0; TN = 20); while every simulation of real stimulation generated an event mark (TP = 20; FN = 0). Tactile and noxious stimulations were therefore detected with 100% sensitivity and specificity.

### 3.3 Clinical validation of the system

**Fig. 3:**
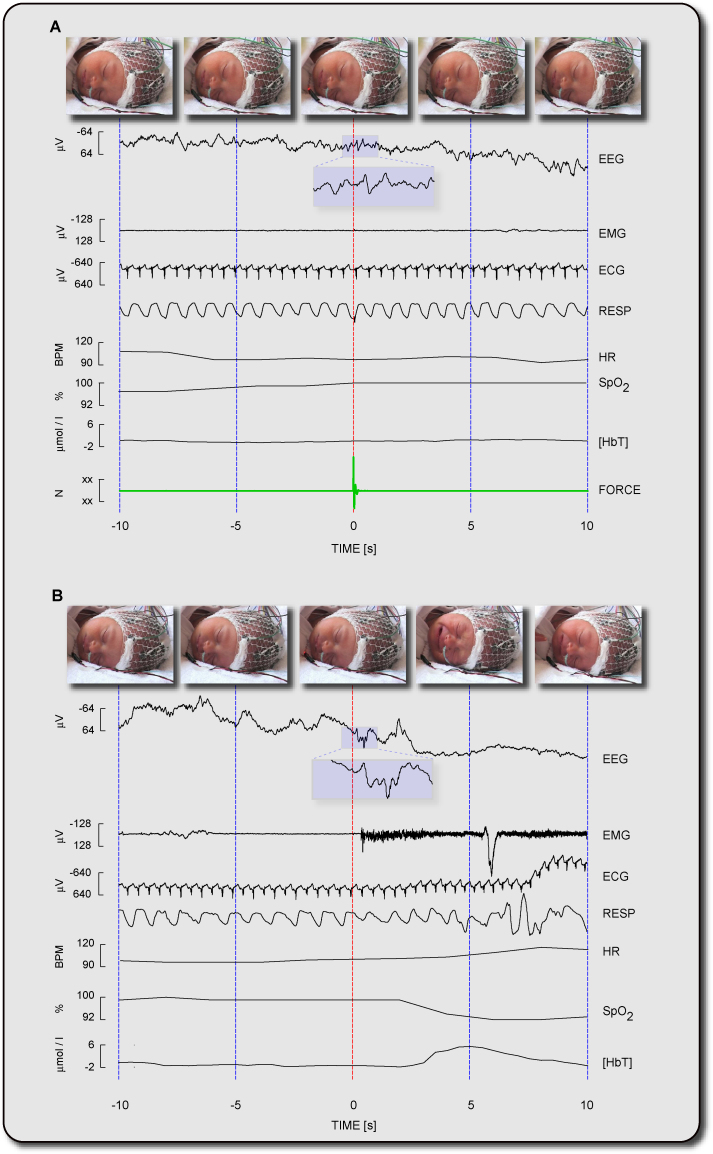
Simultaneous and synchronised acquisitions of video (behavioural activity); EEG (CPz only), total haemoglobin concentration [HbT] and EMG (central nervous system activity); respiration, heart rate, ECG and oxygen saturation (autonomic activity) in correspondence of non-noxious touch (A) and noxious lance of the heel (B). (*Note*: example traces were not all recorded in the same infant.)

Simultaneous and synchronised acquisitions of the CNS, autonomic and behavioural responses following non-noxious touch (Fig. 3A) and noxious lance of the heel (Fig. 3B) were successfully achieved. Differences in the responses to non-noxious and noxious stimulation can be identified from each physiological recording. In the EEG, event-related potentials can be recognised at electrode site CPz. An early component can be seen following both tactile and noxious stimulation, while a later component can be seen only following noxious stimulation. This is coupled with a larger increase in the total haemoglobin concentration in the contralateral somatosensory area as detected by NIRS. Furthermore, from the EMG, video and pulse oximetry it can be seen that noxious lance elicits a larger flexion withdrawal reflex and causes behavioural responses (such as eye squeeze, nasolabial frowning and brow bulging), increase in heart rate and decrease in oxygen saturation which do not follow non-noxious touch.

**Fig. 4:**
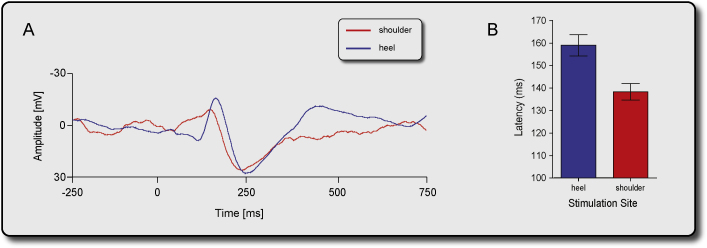
(A) ERP elicited by tapping the shoulder or the heel; (B) the latency of the ERP elicited by tapping the shoulder is shorter than by tapping the heel.

The average ERP elicited by tapping the shoulder was 20.7 ± 15.7 ms earlier than that elicited by tapping the heel (Fig. 4). This is a physiologically plausible result, given the shorter conduction distance between the shoulder and the recording site compared to that from the heel.

## 4 Discussion

This work has shown the development and use of a novel integrated system to study event-locked physiological responses to non-noxious and noxious mechanical stimulation in human infants. The designed method allows simultaneous monitoring of brain activity, reflex withdrawal activity, behavioural activity and autonomic activity in response to sensory stimuli. Our method has the main advantage of permitting the study of pain development in human infants in a non-invasive and ethical way. Furthermore, the heel lance procedures used on the neonatal unit are not changed when a study is undertaken and nursing staff can perform heel lances following routine protocols.

The event-detection interface has an overall precision of 500 μs and accuracy of 460 μs and has a sensitivity and specificity of 100%. The system successfully monitored differences in the physiological responses following tactile or noxious stimulation and latency variations due to difference in conduction distances between the site of stimulation and the brain.

Besides allowing comparison between more direct neuronal recordings with more clinically traditional behavioural and autonomic assessments, this integrated approach gives a more complete picture of the tactile and noxious experience in the human infants. This is extremely important in this pre-verbal population.

In summary, the method described meets the ethical and scientific requirements of pain investigation in human infants. The nursing staff and parents adapted well to the procedure and the infants readily tolerated the handling required. Results were reproducible and the device was able to differentiate responses to nociceptive stimuli from responses to non-nociceptive stimuli. Subsequently, the device has been used effectively on more than 100 test occasions. The proposed method paves the way to improve pain management of newborn infants in clinical practice, enabling improved study of the development of nociception in humans.

## References

[bib0005] Bromm B., Scharein E. (1982). Principal component analysis of pain-related cerebral potentials to mechanical and electrical stimulation in man. Electroencephalogr Clin Neurophysiol.

[bib0010] Duhn L.J., Medves J.M. (2004). A systematic integrative review of infant pain assessment tools. Adv Neonatal Care.

[bib0015] Fitzgerald M., Walker S.M. (2009). Infant pain management: a developmental neurobiological approach. Nat Clin Pract Neurol.

[bib0020] Hrbek A., Hrbkova M., Lenard H.G. (1968). Somato-sensory evoked responses in newborn infants. Electroencephalogr Clin Neurophysiol.

[bib0025] Hrbek A., Karlberg P., Olsson T. (1973). Development of visual and somatosensory evoked responses in pre-term newborn infants. Electroencephalogr Clin Neurophysiol.

[bib0030] Iannetti G.D., Zambreanu L., Cruccu G., Tracey I. (2005). Operculoinsular cortex encodes pain intensity at the earliest stages of cortical processing as indicated by amplitude of laser-evoked potentials in humans. Neuroscience.

[bib0035] IASP Task Force on Taxonomy, Merskey H., Bogduk N. (1994). Part III: pain terms, a current list of definitions and notes on usage. Classification of chronic pain.

[bib0040] McCaffrey M., Pasero C. (1999). Pain: clinical manual.

[bib0045] Rainville P. (2002). Brain mechanisms of pain affect and pain modulation. Curr Opin Neurobiol.

[bib0050] Schnitzler A., Ploner M. (2000). Neurophysiology and functional neuroanatomy of pain perception. J Clin Neurophysiol.

[bib0055] Slater R., Cantarella A., Franck L., Meek J., Fitzgerald M. (2008). How well do clinical pain assessment tools reflect pain in infants?. PLoS Med.

[bib0060] Slater R., Worley A., Fabrizi L., Roberts S., Meek J., Boyd S. (2010). Evoked potentials generated by noxious stimulation in the human infant brain. Eur J Pain.

[bib0065] Takeda M., Takahashi M., Nasu M., Matsumoto S. (2010). In vivo patch-clamp analysis of response properties of rat primary somatosensory cortical neurons responding to noxious stimulation of the facial skin. Mol Pain.

[bib0070] Tracey I., Mantyh P.W. (2007). The cerebral signature for pain perception and its modulation. Neuron.

[bib0075] Treede R.D., Kenshalo D.R., Gracely R.H., Jones A.K. (1999). The cortical representation of pain. Pain.

